# Toxicogenomics profiling of bone marrow from rats treated with topotecan in combination with oxaliplatin: a mechanistic strategy to inform combination toxicity

**DOI:** 10.3389/fgene.2015.00014

**Published:** 2015-02-12

**Authors:** Myrtle Davis, Jianying Li, Elaine Knight, Sandy R. Eldridge, Kellye K. Daniels, Pierre R. Bushel

**Affiliations:** ^1^Toxicology and Pharmacology Branch, Division of Cancer Treatment and Diagnosis, National Cancer InstituteBethesda, MD, USA; ^2^Kelly Government Solutions, Research Triangle ParkNC, USA; ^3^Microarray and Genome Informatics Group, National Institute of Environmental Health Sciences, Research Triangle ParkNC, USA; ^4^Toxicology and Pathology Services, Southern Research InstituteBirmingham, AL, USA; ^5^Biostatistics Branch, Division of Intramural Research, National Institute of Environmental Health Sciences, Research Triangle ParkNC, USA

**Keywords:** toxicogenomics, bone marrow, topotecan, combination, oxaliplatin, enhanced toxicity, EPIG

## Abstract

Combinations of anticancer agents may have synergistic anti-tumor effects, but enhanced hematological toxicity often limit their clinical use. We examined whether “microarray profiles” could be used to compare early molecular responses following a single dose of agents administered individually with that of the agents administered in a combination. We compared the mRNA responses within bone marrow of Sprague-Dawley rats after a single 30 min treatment with topotecan at 4.7 mg/kg or oxaliplatin at 15 mg/kg alone to that of sequentially administered combination therapy or vehicle control for 1, 6, and 24 h. We also examined the histopathology of the bone marrow following all treatments. Drug-related histopathological lesions were limited to bone marrow hypocellularity for animals dosed with either agent alone or in combination. Lesions had an earlier onset and higher incidence for animals given topotecan alone or in combination with oxaliplatin. Severity increased from mild to moderate when topotecan was administered prior to oxaliplatin compared with administering oxaliplatin first. Notably, six patterns of co-expressed genes were detected at the 1 h time point that indicate regulatory expression of genes that are dependent on the order of the administration. These results suggest alterations in histone biology, chromatin remodeling, DNA repair, bone regeneration, and respiratory and oxidative phosphorylation are among the prominent pathways modulated in bone marrow from animals treated with an oxaliplatin/topotecan combination. These data also demonstrate the potential for early mRNA patterns derived from target organs of toxicity to inform toxicological risk and molecular mechanisms for agents given in combination.

## Introduction

Effective anticancer treatments generally require the use of a combination of drugs. A challenge for combination treatment is a shift in the extent and severity of adverse effects for the combination compared with either of the agents when given alone. Most Phase I trials for targeted agent combinations have been designed to escalate the drug doses to the maximally tolerated dose (MTD). Data that are now available suggest that combinations of molecularly targeted agents (MTAs) can lead to more severe toxicities and that dose reduction is often required for regimens to be tolerable (Kummar et al., 2010). Although empirical approaches are currently being used, a preclinical assessment that can provide a mechanistic basis for adverse effects of drug combinations is desirable. Systems-based computational approaches that integrate, transcriptomics, proteomics, or other profiling data may be useful to discover network-level alterations in normal cell signaling in normal tissues after *in vivo* administration.

Toxicogenomics, the application of transcription profiling to toxicology (Nuwaysir et al., 1999), has been widely used for elucidating the molecular and cellular actions of drugs and chemicals on biological systems, flagging potential for toxicity (before functional damages occur), and providing classification of known or new toxicants based on signatures of gene expression (Guerreiro et al., 2003; Ruepp et al., 2005; Bushel et al., 2007; Chengalvala et al., 2007; Ganter et al., 2008; Ryan et al., 2008). In the current study, we examined the benefit of applying transcriptomics to assess risk of enhanced tar-get organ toxicity when two drugs are used in combination. We hypothesized that an additive toxicity pattern can be inferred from comparative analysis of early mRNA responses of tissues obtained following a single dose of two agents given individually with the agents given in combination. We also anticipate that biological pathways revealed by gene response patterns will provide a more comprehensive understanding of toxicity and provide a mechanistic basis for experimental investigation.

For this initial proof of concept, we used the chemotherapeutic agents topotecan and oxaliplatin, which have been explored for use in combination for treating various cancers (Alexandre et al., 2002; Tortora et al., 2002; Elkas et al., 2007). Topotecan is a topoisomerase I (Topo I) inhibitor that forms a complex on DNA leading to double-stranded DNA breaks and ultimately cell death. Oxaliplatin is a diaminocyclohexane platinum compound that acts as a DNA alkylating agent. Pre-exposure to oxaliplatin transiently increased Topo I-mediated single-strand breaks, suggesting that DNA platination might stimulate Topo I DNA cleavage activity providing a rationale for the use of platinum-based compounds with topoisomerase inhibitors (Goldwasser et al., 1999). Combinations of topo I inhibitors and platinum derivatives, in general, have synergistic anti-tumoral effects, but their clinical use is limited by hematological toxicity, which is somewhat dependent on the sequence of drug administration. We also wanted to examine the possibility that the acute response of a target organ to a single dose of treatment and obtaining an associated molecular footprint, can aid in investigation about mechanisms of target-mediated toxicity. In our study, a single dose of topotecan was given to Sprague Dawley rats either before or after administration of oxaliplatin to determine whether combination administration or the order of administration influenced bone marrow toxicity and the microarray signature profile obtained following combined administration of the drugs. We found that a single dose intravenous infusion of topotecan or oxaliplatin, given alone or in combination, to male rats resulted in the formation of histopathological lesions in bone marrow (hypocellularity) observed after only1 h of topotecan administration or 6 h after oxaliplatin administration. The severity of bone marrow lesions increased when topotecan was given prior to oxaliplatin compared with oxaliplatin given prior to topotecan indicating that severity of toxicity was affected by sequence of administration.

We also characterized the molecular and pathway signatures in bone marrow for a topotecan/oxaliplatin combination based on global gene expression analyses and comprehensive bioinformatics profiling. Based on our results, we propose that single dose rodent studies and microarray analysis of mRNA patterns derived from bone marrow represents a mechanistic approach to evaluate the potential risk of enhanced toxicity for combination agents.

## Materials and methods

### Test articles

Oxaliplatin (1.2 g; NSC 266046) and topotecan (310 mg; NSC 609699) were supplied by the National Cancer Institute (NCI) and received at Southern Research Institute on October 19, 2010. The test articles were received on dry ice and then refrigerated. The following reagents were received from the NCI chemical repository and stored at room temperature and used in the preparation of dose formulations: 5% dextrose injection, USP (PSS World Medical; Kennesaw, GA); Saline Solution 0.9% (saline; Nova Tech Inc.; Grand Island, NE).

### Dose formulation preparation

Dose formulations of topotecan were prepared in saline to contain a nominal concentration of topotecan hydrochloride of 1.2 mg/mL. For preparation, the appropriate amount of saline was added to the required amount of topotecan hydrochloride and the formulation was stirred until a solution was obtained. Dose formulations of oxaliplatin were prepared in 5% dextrose in water (D5W) to contain a nominal concentration of oxaliplatin of 3.75 mg/mL. For preparation, the appropriate amount of D5W was added to the required amount of oxaliplatin and the formulation was stirred until a solution was obtained.

### Animals and treatments

The 96 Sprague-Dawley male rats used in this study were obtained from Charles River Laboratories, Inc. (Raleigh, NC). Each rat was procured with an indwelling femoral vein cannula. Animals were given a unique identification number by ear punch. Prior to dosing on day 1, the catheter of each rat was checked for patency. Rats with patent catheters were randomly assigned to one of 8 treatment groups. On day 1, the rats were approximately 8 weeks of age and weighed between 271.2 and 342.2 g. Teklad Certified Rodent Diet 2016C (Harlan; Madison, WI) and tap water (Birmingham public water supply) were provided *ad libitum* to the rats prior to and throughout the study. The animals were individually housed in solid-bottom polycarbonate cages on stainless steel racks in a room maintained at a temperature of 70–79°F and a relative humidity of 44–66%. Room lights were controlled by an automatic timer set to provide a 12/12 light:dark cycle. Heat-treated hardwood chip bedding (P. J. Murphy Forest Products, Corp.; Montville, NJ) was used as bedding material. No known contaminants were present in the food, water, or bedding that would be expected to interfere with or affect the outcome of the study. Cage size and animal care conformed to the guidelines of the Guide for the Care and Use of Laboratory Animals (National Research Council, 2011) and the U.S. Department of Agriculture through the Animal Welfare Act (Public Law 99-198).

The study design (with doses administered) is provided in Table 1. A dose slightly below the MTD was chosen for these studies. Since the goal of our study was to investigate gene responses induced by a single dose that could potentially be premonitory for toxicity, we administered a dose that would not be confounded by marked pathological changes in the tissue. The MTD for oxaliplatin in rats was referenced in the Center for Drug Evaluation and Research pharmacology review of NDA # 21-492 (FDA, 2002) and is close to the human clinical dose of 85 mg/m^2^. The dose selected for topotecan was derived from the time course of the hematological effects of topotecan after two consecutive daily administrations to tumor-bearing rats that was described using a semiphysiological model to predict the hematotoxic effects of topotecan (Friberg et al., 2002; Segura et al., 2004). Each rat was administered a single intravenous (IV) infusion dose, given over a 30 min interval, of topotecan (of 4.7 mg/kg), oxaliplatin (15 mg/kg), or respective vehicle control formulation (see Table 1). Thirty minutes after the end of the first infusion, topotecan, oxaliplatin, or a respective vehicle control formulation was administered by IV infusion, given over a 30 min interval. For example, animals given topotecan (or corresponding vehicle) during the first infusion were given oxaliplatin (or corresponding vehicle) during the second infusion. All doses were delivered at a flow rate of approximately 4 mL/kg/30 min. Infusion volumes were based on the mean body weight of animals on the day prior to dosing. Rats were observed twice daily during the pre-study and study periods for signs of mortality and morbidity. Detailed clinical examinations of each rat were collected prior to euthanasia. Each animal was weighed on the day of dosing prior to dose administration (for the calculation of infusion rate; data not reported).

**Table 1:** **Study design**.

**Group**	**Treatment**	**Dose of topotecan (mg/kg)**	**Dose of oxaliplatin (mg/kg)**	**No. animals per group**	**No. animals per necropsy time point (Hours)**		
					**1**	**6**	**24**
1	0.9% NaCl(30 min infusion) followed by D5W (30 min infusion)	0	0	12	4	4	4
2	D5W(30 min infusion) followed by 0.9% NaCl(30 min infusion)	0	0	12	4	4	4
3	Topotecan (30 min infusion) followed by D5W (30 min infusion)	4.7 (first infusion)	0	12	4	4	4
4	Oxaliplatin (30 min infusion) followed by 0.9% NaCl (30 min infusion)	0	15 (first infusion)	12	4	4	4
5	Topotecan (30 min infusion) followed by Oxaliplatin (30 min infusion)	4.7 (first infusion)	15 (second infusion)	12	4	4	4
6	Oxaliplatin (30 min infusion) followed by Topotecan (30 min infusion)	4.7 (second infusion)	15 (first infusion)	12	4	4	4
7	0.9% NaCl(30 min infusion) followed by Oxaliplatin (30 min infusion)	0	15 (second infusion)	12	4	4	4
8	D5W(30 min infusion) followed by Topotecan (30 min infusion)	4.7 (second infusion)	0	12	4	4	4

### Histopathology

Samples of femur bone marrow were collected for histopathology and fixed in 10% neutral buffered formalin. All slides were examined by a veterinary pathologist. Each lesion was listed and coded by the most specific topographic and morphologic diagnoses, severity, and distribution using Toxicology Data Management System (TDMS) nomenclature. A four-step grading system was used to rank the severity of microscopic lesions for comparison among groups.

### Tissue collection and RNA isolation

At 1, 6, or 24 h after the completion of dosing, 4 rats per group were euthanized by CO_2_ asphyxiation. Immediately after euthanasia, bone marrow from left femurs was collected for RNA isolation. Bone marrow was flushed from bone using RNAlater® and refrigerated (approximately 2–8°C) for at least 24 h and then stored at or below −20°C.

RNA isolation from individual tissues was accomplished using an RNeasy microarray kit (QIAGEN Inc., Valencia, CA). After extraction, the RNA concentration of each sample was determined using a RiboGreen assay. The RNA Integrity Number (RIN) of each sample was determined using an Agilent 2100 Bioanalyzer with 2100 Expert Software (Version B.02.06.S1418; Agilent, Santa Clara, CA). Only samples with a RIN of 7.5 or higher were deemed acceptable for gene expression analysis. Two samples, one from group 3 and a group 2 did not meet the RIN for further processing. Each of the remaining RNA samples were diluted to the required concentration (250 ng/μ L) and then stored at or below −70°C prior to shipment to Expression Analysis (Durham, NC) for microarray hybridization.

### Microarray hybridization

Microarray data was generated by Expression Analysis (Durham, NC). RNA samples were converted into labeled target antisense RNA (cRNA) using the Single-Round RNA Amplification and Biotin Labeling System (Enzo Life Sciences, Farmingdale, NY). Briefly, 2.5 μ g of total RNA was converted into double stranded cDNA via reverse transcription using an oligo-d(T) primer-adaptor. This cDNA was purified and used as a template for *in vitro* transcription using T7 RNA polymerase and biotinylated ribonucleotides. The resulting cRNA was purified using magnetic beads and quantitated using spectrophotometry. Next, 11 μ g of purified cRNA was fragmented using a 5X fragmentation buffer (200 mM Tris-Acetate, pH 8.1, 500 mM KOAc, 150 mM MgOA), then a hybridization cocktail was prepared and added to the fragmentation product using the Hybridization, Wash and Stain kit (Affymetrix, Santa Clara, CA), applied to Affymetrix GeneChip Rat Genome 230 2.0 Arrays, and incubated at 45°C for 16 h. Following hybridization, arrays were washed and stained using standard Affymetrix procedures before scanning on the Affymetrix GeneChip Scanner 3000 using factory PMT settings. Data extraction was completed with Expression Console software using a target scaling of 500. The data are available in the Chemical Effects in Biological Systems (CEBS) repository (Waters et al., 2008) under investigation accession number: 007-00005-0000-000-2 and study accession number: 007-00005-0001-000-3 as well as in the Gene Expression Omnibus (GEO) (Edgar et al., 2002; Barrett et al., 2013) under accession number GSE63902.

### Detection of differentially expressed genes (DEGs)

Data analysis was performed using the Bioconductor R package (Gentleman, 2005). We used the package “affyQCReport,” combined with principal component analysis (PCA), for array quality control (i.e., outliers detection). After the one outlier sample was excluded (sample 1344-05H, 6 h oxaliplatin followed by vehicle), the remaining Affymetrix raw CEL files were preprocessed using the robust multichip average (RMA) algorithm (Irizarry et al., 2003a,b); which includes background correction, quantile normalization, and summarization by the median polish approach. Next, we performed PCA-based gene filtering on the log base 2 scale data from RMA using the package “pvac,” where the filtering is based on a score measuring consistency among probes within a probe set (Lu et al., 2011). Finally, the statistical significance of DEGs was accessed by comparison of treated samples to time-matched controls using the empirical Bayes based method known as a limma *t*-test, which is available in the bioconductor package limma (Smyth, 2005). Analyses were carried out in two batches, where the first batch includes samples from groups 1, 3, 5, and 7, and the second batch contains samples from groups 2, 4, 6, and 8, (see study design Table 1).

### Detection of patterns of co-expressed genes

log base 2 scaled data of the probes remaining after the PCA-based filtering were analyzed for expression patterns across the samples using methodology known as extracting gene expression patterns and identifying co-expressed genes (EPIG) (Chou et al., 2007). For each treatment group, the pixel intensity data for each probe was converted to a ratio value by dividing the average probe pixel intensity by the 1 h control samples from that group and then taking the log base 2. EPIG uses correlations (*r*) across all the sample groups, signal to noise ratio (*s*/*n*) within groups of samples, and magnitude of fold change (FC) for a probe within a group to first detect all potential patterns in the data, and then categorize each probe to the pattern that is most statistically significant in terms of the correlation between the probe profile and the pattern. The parameter settings for the EPIG analysis were the defaults: *r* = 0.8, *s*/*n* = 2.5, and *FC* = 0.5. We used a minimum pattern cluster size of 6 for finding all potential patterns.

### Enrichment of biological processes based on DEGs

For each DEGs list derived at *p* < 0.01 and absolute fold change >2, the Affymetrix GeneChip Rat Genome 230 2.0 Array probe sets were mapped to the Gene Ontology (GO) biological processes (BPs) of the genes they represent using version 2.10.1 of the GO database and the rat2302 database. The 2.14.0 version of the “topGO” Bioconductor package in R was used to perform enrichment of GO BP terms. The classic algorithm (where the significance of a node is considered independent of the significance of the neighboring nodes) and the Kolmogorov-Smirnov test statistic were used for enrichment with node size of at least 5 DEGs. Significance of enrichment was set to *p* < 0.05. The union of the enriched GO BPs terms from all of the DEGs lists yielded 641 terms. BP terms which were not significant had missing *p*-values and were imputed with 1.0. The 641 GO BPs terms were clustered based on the −log base 10 *p*-values and using Pearson correlation (*r*) as the dissimilarity metric with average linkage grouping. Clusters (those with a 1- *r* ≥ 0.9) of GO BP terms (*n* ≥ 30) were labeled according to the node having the maximum number of paths to it within the GO BP subtree directed acyclic graph derived from the terms in the cluster.

### Pathway analysis of genes within patterns extracted by EPIG

Clusters of genes identified by EPIG were analyzed using Pathway Studio 9® (Ariadne Genomics, Rockville, MD), to find enriched pathways. Enrichment analysis in Pathway Studio 9® was performed by Gene Set Enrichment Analysis (GSEA) and Sub-Network Enrichment Analysis (SNEA) algorithms. Functional enrichment was performed using Fisher's exact test. SNEA enrichment in Pathway Studio was calculated using the Mann-Whitney test, a non-parametric method for comparing the medians of two distributions. Significant enrichment was set at *p* < 0.05. For GSEA, pathways with fewer than 3 entities represented were filtered from the data sets.

### Visualization of gene expression on pathways

To visualize the changes of gene expression within a particular pathway, Pathvisio version 3.1.3 (Van Iersel et al., 2008) was used to overlay the average of the log base 2 ratio values from the replicate samples onto biological pathways obtained from Wikipathways (Kelder et al., 2012). The cluster IDs from UniGene (according to the rat Rn4 July 12, 2012 release of refSeq version 54) was used to map the probe sets on the microarray to the genes on the Wikipathways.

### Gene regulatory network reconstruction

The Gene Regulatory Network Inference (GRNInfer) software (Wang et al., 2006) with default parameter settings (λ = 0.0 and threshold = 1 × 10^−6^ controlling the sparseness and the complexity of the network respectively) was used to reconstruct the interactions of the 19 genes in the p53 signaling Wikipathway that had gene expression data mapped. The gene expression data from microarray probes mapping to the same UniGene cluster were averaged and then biological replicates at each time point were averaged. GRNInfer uses linear programming and singular value decomposition of the time point gene expression data to derive of the interactions of the genes within the network given the above thresholds. For topotecan followed by oxaliplatin and oxaliplatin followed by topotecan, the averaged gene expression measures at each time point were used to reconstruct the gene regulatory network depending of the order of administration of the two drugs.

## Results

### Clinical observations

No mortality occurred during the study and no drug- or treatment-related adverse clinical signs were observed for any animal during the study. The mean body weight of animals in the individual dose groups ranged from 300 to 310 g on the day of dosing.

### Histopathology

**Figure 1:**
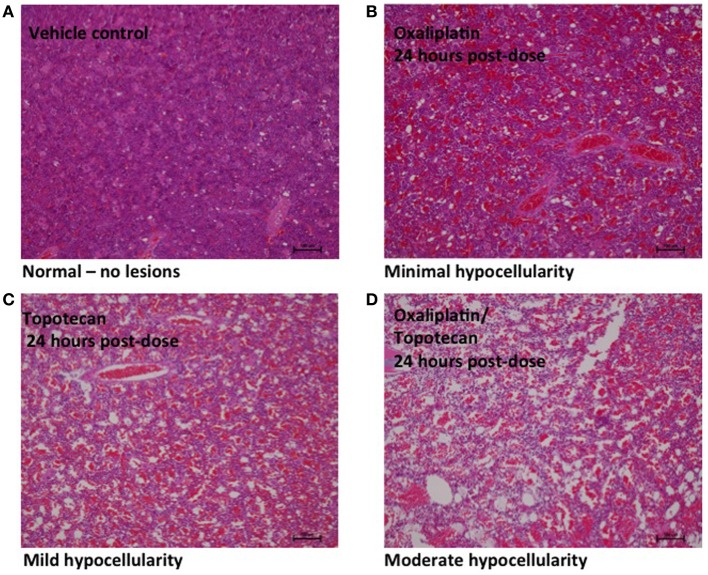
**Representative images of bone marrow hypocellularity in femur from male rats 24 h after a single 30 min IV infusion dose of oxaliplatin, topotecan or combination of oxaliplatin followed by topotecan**. Hematoxylin and eosin stained tissue sections; 10X objective; bars = 100 μm. **(A)** Male rat exposed to vehicle control. **(B)** Minimal hypocellularity in male rat exposed to oxaliplatin. **(C)** Mild hypocellularity in male rat exposed to topotecan. **(D)** Moderate hypocellularity in male rat exposed to oxaliplatin followed by topotecan. Bone marrow hypocellularity was graded based on the estimated percentage of cell loss with minimal hypocellularity = 10–20% cell loss, mild hypocellularity = 30–40% cell loss, moderate hypocellularity = 50–60% cell loss, and marked hypocellularity = greater than 60% cell loss.

Bone marrow hypocellularity was observed in the bone marrow of animals dosed with topotecan, oxaliplatin, or a combination of these two drugs (Figure 1 and Table 2). Minimal bone marrow hypocellularity consisted of an approximately 10–20% decrease in the normal population of cells which reside in the bone marrow in comparison with control animals. The remaining cell population consisted primarily of band and mature granulocytes (neutrophils and eosinophils), mature erythrocytes, and megakaryocytes. Occasionally apoptotic cells were observed in the bone marrow of some animals. These lesions were observed as early as 1 h after the end of topotecan administration and 6 h after the end of oxaliplatin administration. Bone marrow hypocellularity was observed at a higher and earlier onset of incidence in animals that received topotecan alone or topotecan in combination with oxaliplatin than in dose groups that received only oxaliplatin. One-hour post-treatment, bone marrow hypocellularity was observed for animals in all of the dose groups administered topotecan alone or topotecan in combination with oxaliplatin, but not in the dose groups given only oxaliplatin. There also appeared to be an increase in severity of bone marrow suppression from mild to moderate when topotecan was given prior to oxaliplatin than when oxaliplatin was given prior to topotecan. These data support a sequence dependency for the severity of bone marrow toxicity for this combination.

**Table 2:** **Histopathology of the bone marrow**.

**1 h POST-DOSE: SEVERITY OF TEST ARTICLE-RELATED MICROSCOPIC LESIONS**								
Tissue, microscopic lesion	0.9%saline D5W	D5W 0.9% saline	Topotecan/ D5W	Oxaliplatin 0.9% saline	Topotecan Oxaliplatin	Oxaliplatin Topotecan	0.9% saline oxaliplatin	D5W/ Topotecan
Bone marrow, hypocellularity	NA	NA	Minimal to mild	NA	Minimal	Minimal to mild	NA	Minimal
**6 h POST-DOSE: SEVERITY OF TEST ARTICLE-RELATED MICROSCOPIC LESIONS**								
Tissue, microscopic lesion	0.9%saline D5W	D5W 0.9% saline	Topotecan D5W	Oxaliplatin 0.9% saline	Topotecan Oxaliplatin	Oxaliplatin Topotecan	0.9% saline oxaliplatin	D5W Topotecan
Bone marrow, hypocellularity	NA	NA	Minimal to mild	Minimal to mild	Minimal to mild	Mild	Minimal to mild	Minimal to mild
**24 h POST-DOSE: SEVERITY OF TEST ARTICLE-RELATED MICROSCOPIC LESIONS**								
Tissue, microscopic lesion	0.9%saline D5W	D5W 0.9% saline	Topotecan D5W	Oxaliplatin 0.9% saline	Topotecan Oxaliplatin	Oxaliplatin Topotecan	0.9% saline oxaliplatin	D5W Topotecan
Bone marrow, hypocellularity	NA	NA	Mild	Minimal to mild	Mild to moderate	Mild to moderate	Minimal to mild	Mild to moderate

### RNA isolation

The integrity of each isolated RNA sample was equal to or greater than the protocol-specified minimum RIN (7.5), with the exception of two samples. Acceptance criteria could not be obtained for bone marrow collected from one of the animals from group 3 (topotecan/D5W treated group) and one animal in group 2 (D5W/saline vehicle control group). These samples were not submitted for gene expression analysis.

### Patterns of co-expressed genes

**Figure 2:**
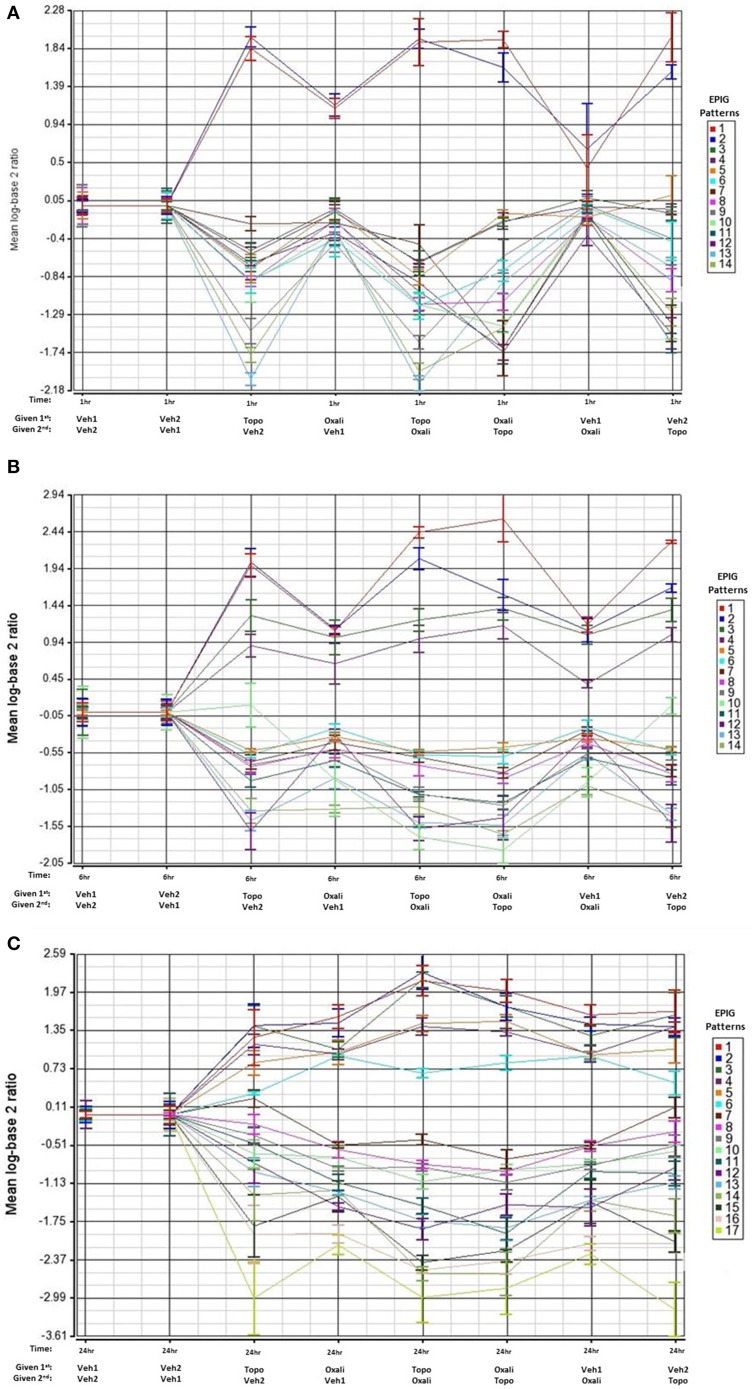
**EPIG patterns across treatments groups 1, 6, and 24 h post-treatment**. The average of the log base 2 ratio (treated sample to the average of the time-matched control) from the top 6 expression profiles with the highest degree of correlation are plotted and displayed on the y-axis. The x-axis contains the treatments and the order of exposure. Oxali = oxaliplatin (15 mg/kg) and Topo = topotecan (4.7 mg/kg). **(A)** Patterns extracted by EPIG of mRNA from bone marrow samples that were collected 1 h post-treatment administration. The row labeled 1st denotes which agent was administered first. **(B)** Same as A except analysis of mRNA from bone marrow samples that were collected 6 h post-treatment. **(C)** Same as A except analysis of mRNA from bone marrow samples collected 24 h post-treatment.

We focused our analysis on uncovering groups of genes displaying similarity in their expression patterns and comparing the gene responses between treatment groups. The EPIG approach utilizes the underlying structure of gene expression data to extract patterns and identify co-expressed genes that are responsive to experimental conditions (Chou et al., 2007). The response patterns of genes were used to select gene sets that represent potential signatures for effects of the combination treatments that differed from that of the single agents. As shown in Figures 2A–C, several patterns of gene expression are induced or repressed relative to time-matched controls at the 1-, 6-, or 24 h time points respectively. Patterns that identified differences between individual treatment and combination treatment groups are marked with an asterisk in Supplementary Figures 1–3. The Venn diagrams in Supplementary Figure 4 reveal that there is little overlap of co-expressed genes changes (of 4-fold or more) between oxaliplatin and topotecan except for at the 24 h time point where 73 genes overlap.

**Figure 3:**
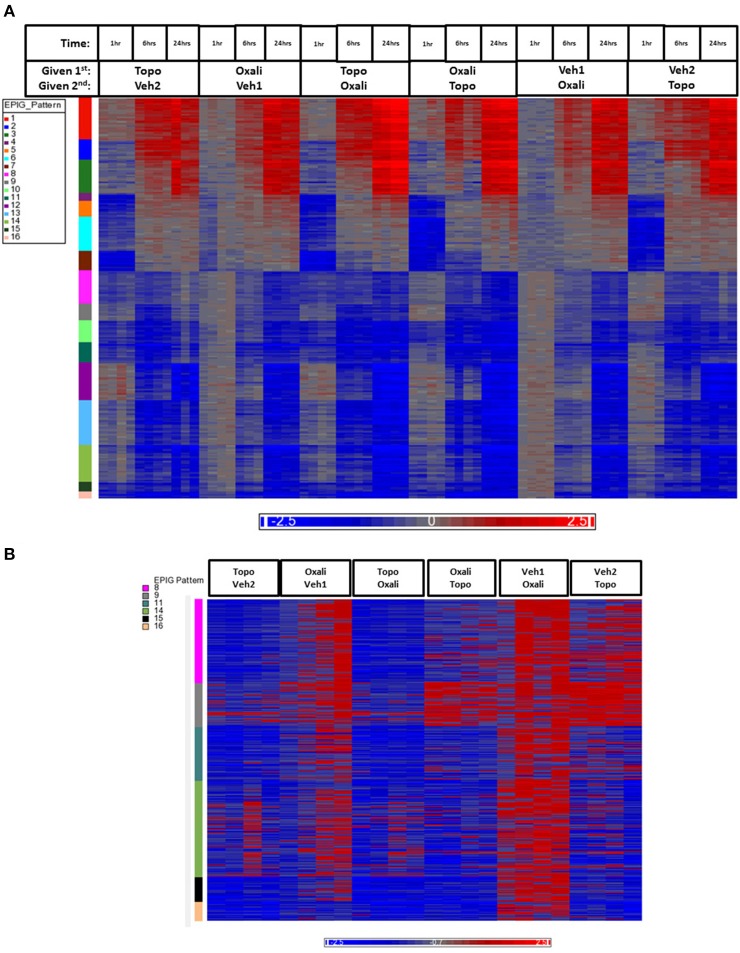
**Heatmap of gene expression data analyzed by EPIG**. **(A)** The 1393 gene probes categorized to the 16 EPIG patterns (y-axis color column) across the 1, 6, and 24 h time points are in rows and the samples are in columns. **(B)** Patterns 8, 9, 11, 14–16 from the samples at just the 1 h time point. The labeling of samples is according to the time and order in which the agent was given (either 1st or second). Oxali is oxaliplatin (15 mg/kg) and Topo = topotecan (4.7 mg/kg). Veh1 is the vehicle used for Oxali, and Veh2 is the vehicle used for Topo. The data is the log base 2 ratio (treated sample to the average of the time-matched control) and the scale on the bottom displays the color range for the log base 2 ratio values. Red denotes upregulation, blue downregulation, and gray relatively no change.

The heat map in Figure 3A shows gene expression patterns across all treatment groups and time points. There is a clear time response in the patterns of gene expression from the treatments where by the 24 h time point, the patterns of expression are either maximally induced or maximally repressed. A list of representative pattern-specific genes showing at least 4-fold differences from their respective controls at the 24 h bone marrow collection time point is provided in Table 3 (up-regulated) and Table 4 (down-regulated). Notably, several genes related to chondrogenesis, bone repair, and differentiation were profoundly increased in response to combination treatment when compared to individual treatments. As show in the visualization of just the 1 h time points (Figure 3B), the co-expressed genes in patterns 8, 9 and pattern 11 vary depending on whether topotecan was given before or after the vehicle or oxaliplatin. On other hand, the co-expressed genes in patterns 14–16, vary depending on whether oxaliplatin was given before or after the vehicle. The 244 probes in patterns 8, 9, and 11, representing 59 co-expressed genes, enrich for pathways related to mRNA splicing, splicesomes, metabolism, cell cycle and DNA replication. The 188 probes in patterns 14–16, representing 45 co-expressed genes, enrich for pathways related to chromosome organization, chromatin packaging and remodeling. Full lists of genes derived from each pattern with absolute fold change of 4 or greater from their respective controls and *p* < 0.05, are provided in Supplementary Table 1.

**Table 3:** **List of representative pattern-specific genes showing at least 4-fold increase from the average of their respective controls 24 h after treatment**.

**Probe ID**	**Symbol**	**Entrez gene name**	**Pattern number**	**Control D5W**	**Control NaCl**	**Topotecan followed by D5W**	**Oxaliplatin followed by NaCl**	**Toptecan followed by oxaliplatin**	**Oxaliplatin followed by topotecan**	**NaCl followed by oxaliplatin**	**D5W followed by topotecan**
1368530_at	MMP12	Matrix metallopeptidase 12 (macrophage elastase)	1	0.49	−0.33	11.57	15.11	54.72	31.26	12.98	22.82
1376908_at	IFIT3	Interferon-induced protein with tetratricopeptide repeats 3	5	0.00	−0.33	10.34	10.21	30.05	16.47	5.87	7.95
1367570_at	TAGLN	Transgelin	1	0.00	−0.33	6.92	5.81	17.71	12.09	8.80	9.15
1373401_at	TNC	Tenascin C	1	0.49	0.32	6.80	6.61	8.27	5.43	7.75	5.86
1367712_at	TIMP1	TIMP metallopeptidase inhibitor 1	3	−0.49	0.33	6.51	3.30	14.33	9.40	4.19	8.14
1371310_s_at	SERPINH1	Serpin peptidase inhibitor, clade H (heat shock protein 47)	1	0.50	0.33	5.54	5.21	9.29	7.71	5.97	6.57
1383075_at	CCND1	Cyclin D1	1	0.00	0.33	5.44	5.58	8.49	5.51	3.75	5.80
1376481_at	ADAMTS9	ADAM metallopeptidase with thrombospondin type 1 motif, 9	1	0.49	0.33	5.16	5.92	8.68	8.22	5.02	7.36
1373463_at	COL5A2	Collagen, type V, alpha 2	1	0.49	−0.33	5.14	3.86	6.21	6.08	4.07	4.21
1367973_at	CCL13	Chemokine (C-C motif) ligand 13	3	0.00	0.29	4.74	4.85	17.18	11.17	5.79	6.04
1379285_at	RTP4	Receptor (chemosensory) transporter protein 4	2	0.00	−0.33	4.69	4.38	11.12	6.45	2.87	4.48
1387232_at	BMP4	Bone morphogenetic protein 4	1	−0.50	0.33	4.55	5.68	9.16	9.33	5.62	7.72
1383564_at	IRF7	Interferon regulatory factor 7	2	0.00	0.33	4.53	3.68	9.57	5.05	3.07	3.42
1387197_at	OMD	Osteomodulin	1	0.00	−0.33	4.47	3.53	7.12	4.44	4.24	3.91
1370895_at	COL5A2	Collagen, type V, alpha 2	1	0.49	−0.33	4.43	3.56	5.52	5.28	3.97	3.66
1376845_at	IFI27L2	Interferon, alpha-inducible protein 27-like 2	2	−0.42	−0.33	4.42	7.44	15.15	9.46	4.73	7.69
1367691_at	PRKCDBP	Protein kinase C, delta binding protein	1	0.50	0.33	4.42	5.43	9.95	8.03	5.36	7.07
1367896_at	CA3	Carbonic anhydrase III, muscle specific	1	−0.10	−0.33	4.42	7.77	6.69	8.67	5.15	9.88
1368059_at	CRYM	Crystallin, mu	1	0.50	−0.33	4.32	5.72	8.54	7.70	5.05	7.17
1371643_at	CCND1	Cyclin D1	1	0.00	−0.33	4.26	4.95	7.26	5.49	3.94	5.16
1367562_at	SPARC	Secreted protein, acidic, cysteine-rich (osteonectin)	1	−0.01	0.33	4.26	3.19	5.15	3.47	3.97	2.96
1398270_at	BMP2	Bone morphogenetic protein 2	1	0.00	−0.33	4.18	5.49	9.56	8.44	5.10	5.35
1388569_at	SERPINF1	Serpin peptidase inhibitor, clade F, member 1	1	0.00	0.33	3.85	3.30	5.44	4.30	3.92	3.36
1386912_at	PCOLCE	Procollagen C-endopeptidase enhancer	1	0.00	0.33	3.84	4.51	7.72	6.75	4.38	5.15
1386884_at	HTRA1	HtrA serine peptidase 1	1	0.00	0.33	3.71	3.50	6.86	4.71	3.74	4.66
1388439_at	FKBP10	FK506 binding protein 10, 65 kDa	1	0.00	0.33	3.31	2.87	4.27	3.71	3.22	3.32
1377367_at	KAZALD1	Kazal-type serine peptidase inhibitor domain 1	1	0.00	−0.33	3.30	3.40	5.51	4.99	2.99	4.64
1389918_at	PALLD	Palladin, cytoskeletal associated protein	1	0.01	−0.33	3.28	3.53	5.88	5.98	2.97	3.78
1373674_at	MFAP5	Microfibrillar associated protein 5	1	0.01	0.33	3.27	4.42	4.65	4.51	4.51	5.44
1384180_at	IFIT2	Interferon-induced protein with tetratricopeptide repeats 2	2	0.00	0.33	3.22	4.21	7.22	4.67	2.60	2.59
1386977_at	CA3	Carbonic anhydrase III, muscle specific	1	−0.01	−0.33	3.20	6.20	5.83	7.29	4.91	8.97
1386953_at	HSD11B1	Hydroxysteroid (11-beta) dehydrogenase 1	1	0.00	−0.32	3.19	3.10	4.09	3.23	3.38	4.14
1372219_at	TPM2	Tropomyosin 2 (beta)	1	0.00	−0.32	3.18	3.59	7.20	5.12	4.12	4.06
1387770_at	Ifi27l1	Interferon, alpha-inducible protein 27 like 1	5	−0.50	0.31	3.16	3.91	6.03	4.74	2.67	4.12
1370301_at	MMP2	Matrix metallopeptidase 2 (gelatinase A)	1	0.00	−0.33	3.01	6.32	9.88	10.38	5.08	3.96
1393240_at	EFEMP2	EGF containing fibulin-like extracellular matrix protein 2	1	0.50	0.33	3.00	3.60	5.17	4.64	3.13	4.03
1373210_at	LAMB1	Laminin, beta 1	1	0.00	−0.33	3.00	3.65	6.70	4.69	3.54	3.44
1378443_at	SLAMF9	SLAM family member 9	2	0.00	0.33	2.99	2.63	5.97	3.15	2.17	3.23
1371150_at	CCND1	Cyclin D1	1	−0.50	−0.33	2.53	2.88	4.21	3.45	2.44	3.48
1390192_at	SLC27A3	Solute carrier family 27 (fatty acid transporter), member 3	1	0.00	0.33	2.51	4.51	6.68	6.17	4.42	4.04
1376151_a_at	LOC100365106	rCG32755-like	5	0.00	−0.33	2.50	3.22	5.37	4.85	2.01	2.73
1370927_at	COL12A1	Collagen, type XII, alpha 1	1	0.00	0.33	2.49	2.88	4.25	3.39	2.63	2.69
1369665_a_at	IL18	Interleukin 18 (interferon-gamma-inducing factor)	3	0.00	−0.33	2.49	2.05	4.05	2.57	2.13	2.60
1387259_at	CDH2	Cadherin 2, type 1, N-cadherin (neuronal)	1	0.00	−0.33	2.47	2.95	4.16	4.45	2.61	3.49
1383266_at	SFRP1	Secreted frizzled-related protein 1	1	−0.50	−0.33	2.46	2.66	4.50	2.99	2.68	2.95
1369720_at	MYO1B	Myosin IB	1	0.00	−0.33	2.46	2.79	4.98	4.42	3.66	2.74
1387394_at	IL2RB	Interleukin 2 receptor, beta	5	−0.50	0.33	2.42	2.85	4.26	5.07	2.57	2.99
1397304_at	Igtp	Interferon gamma induced GTPase	5	0.00	0.33	2.40	3.15	5.91	4.46	2.26	2.80
1384182_at	FERMT2	Fermitin family member 2	1	0.00	0.33	2.40	2.92	4.27	4.10	2.91	3.26
1372439_at	COL4A1	Collagen, type IV, alpha 1	1	0.00	0.33	2.40	3.21	5.98	4.10	3.39	3.26
1375377_at	IGSF3	Immunoglobulin superfamily, member 3	1	0.00	0.33	2.39	2.91	4.50	4.43	3.20	3.33
1376706_at	TMEM47	Transmembrane protein 47	1	0.50	−0.33	2.38	3.00	4.58	4.23	2.85	3.15
1386965_at	LPL	Lipoprotein lipase	1	0.00	0.33	2.38	5.53	6.04	6.35	3.96	5.18
1369640_at	GJA1	Gap junction protein, alpha 1, 43kDa	1	0.00	0.33	2.37	2.87	4.74	4.37	3.94	3.43
1387122_at	PLAGL1	Pleiomorphic adenoma gene-like 1	1	0.00	0.33	2.36	2.79	4.26	3.79	2.04	3.04
1387015_at	Pfn2	Profilin 2	1	0.00	0.33	2.36	2.62	5.68	4.42	3.01	3.69
1372064_at	CXCL16	Chemokine (C-X-C motif) ligand 16	3	0.00	−0.33	2.35	2.30	5.27	3.64	2.05	2.52
1368961_at	MMP23B	Matrix metallopeptidase 23B	1	0.50	−0.33	2.35	3.09	5.94	5.17	3.36	3.90
1382192_at	LYVE1	Lymphatic vessel endothelial hyaluronan receptor 1	3	0.00	0.32	2.35	2.60	5.04	4.09	2.23	3.53
1370167_at	SDC2	Syndecan 2	1	0.00	−0.33	2.35	2.69	4.25	3.66	3.11	3.12
1370244_at	CTSL2	Cathepsin L2	1	−0.50	−0.33	2.33	2.45	4.55	3.07	3.22	2.84
1370333_a_at	IGF1	Insulin-like growth factor 1 (somatomedin C)	3	−0.50	−0.33	2.32	1.82	4.08	2.97	2.80	3.18
1376198_at	CLMP	CXADR-like membrane protein	1	0.00	0.33	2.32	2.84	4.28	4.22	2.71	3.36
1368945_at	BMP2	Bone morphogenetic protein 2	1	0.00	0.33	2.32	2.34	4.12	3.09	2.96	2.18
1388856_at	KITLG	KIT ligand	1	0.00	0.32	2.32	2.98	4.05	4.03	3.03	3.46
1391489_at	IRGM	Immunity-related GTPase family, M	2	0.00	0.33	2.31	2.23	5.14	3.41	1.88	2.23
1370959_at	COL3A1	Collagen, type III, alpha 1	1	0.00	0.32	2.31	3.36	5.34	4.36	2.85	3.42
1384392_at	CYP26B1	Cytochrome P450, family 26, subfamily B, polypeptide 1	1	0.00	0.33	2.31	3.28	6.54	5.60	3.08	2.17
1392265_s_at	MMP23B	Matrix metallopeptidase 23B	1	0.00	0.33	2.29	2.49	6.16	4.13	2.81	3.65
1376920_at	LOC500013	Similar to sterile alpha motif domain containing 9-like	5	0.00	0.33	2.27	3.04	4.17	3.63	2.06	2.30
1387455_a_at	VLDLR	Very low density lipoprotein receptor	3	0.00	0.33	2.26	2.04	5.29	3.14	2.69	3.12
1394022_at	ID4	Inhibitor of DNA binding 4, dominant negative helix-loop- helix protein	1	−0.50	−0.33	2.25	2.38	4.11	3.18	2.70	2.21
1387995_a_at	Ifitm3	Interferon induced transmembrane protein 3	2	0.00	−0.33	2.24	2.48	5.15	3.34	2.59	2.32
1385248_a_at	OGN	Osteoglycin	1	0.48	0.33	2.24	3.20	8.01	3.97	4.86	3.37
1387472_at	CD3D	CD3d molecule, delta (CD3-TCR complex)	5	−0.50	−0.33	2.24	2.76	4.40	4.32	2.86	2.95
1377340_at	TFPI2	Tissue factor pathway inhibitor 2	3	0.00	−0.33	2.24	2.54	4.71	2.97	2.73	2.96
1368821_at	FSTL1	Follistatin-like 1	1	−0.50	0.33	2.23	2.61	5.00	4.11	3.62	3.47
1370950_at	PPAP2B	Phosphatidic acid phosphatase type 2B	1	0.00	0.33	2.22	2.99	4.24	3.99	2.88	2.97
1371369_at	COL6A2	Collagen, type VI, alpha 2	1	0.50	0.33	2.20	2.59	4.19	3.50	2.73	2.55
1374496_at	TRIL	TLR4 interactor with leucine-rich repeats	1	0.50	0.33	2.17	2.31	4.83	3.45	2.67	2.49
1389546_at	AMOTL2	Angiomotin like 2	1	0.50	−0.33	2.17	4.01	5.02	5.11	3.38	3.23
1372101_at	PPAP2B	Phosphatidic acid phosphatase type 2B	1	0.00	−0.33	2.17	3.14	4.13	3.85	2.99	2.95
1389651_at	APLN	Apelin	1	−0.50	−0.33	2.16	4.39	8.56	7.82	5.02	2.93
1369207_at	IL7	Interleukin 7	1	−0.50	−0.33	2.00	2.30	4.07	2.88	2.63	2.86
1390075_at	OLFML2B	Olfactomedin-like 2B	1	0.00	−0.33	2.00	3.19	5.15	3.83	3.06	3.20
1372587_at	EMCN	Endomucin	1	0.00	−0.33	1.98	2.43	4.16	2.74	2.99	2.33
1370202_at	PLA2G16	Phospholipase A2, group XVI	3	0.00	−0.32	1.97	2.03	4.05	2.80	2.12	2.31
1368702_at	PAWR	PRKC, apoptosis, WT1, regulator	3	0.50	−0.33	1.96	1.97	4.35	3.14	2.01	2.48
1371349_at	COL6A1	Collagen, type VI, alpha 1	1	0.00	−0.33	1.96	2.33	4.00	3.48	2.58	2.42
1376106_at	TMEM178	Transmembrane protein 178	1	0.00	0.33	1.96	2.84	5.12	4.75	2.60	3.70
1384558_at	Plac9	Placenta-specific 9	1	0.50	0.33	1.93	2.55	4.07	2.73	2.33	3.40
1390776_at	IRX3	Iroquois homeobox 3	1	0.00	−0.33	1.93	2.18	4.03	3.30	2.17	2.34
1390156_a_at	PRICKLE2	Prickle homolog 2 (Drosophila)	1	0.00	−0.33	1.89	2.78	4.27	4.35	2.42	2.99
1373245_at	COL4A1	Collagen, type IV, alpha 1	1	0.00	−0.33	1.88	2.63	5.20	3.83	2.87	2.55
1385173_at	EBF3	Early B-cell factor 3	1	0.00	0.33	1.82	3.27	4.81	5.02	3.12	3.58
1368271_a_at	FABP4	Fatty acid binding protein 4, adipocyte	1	0.00	−0.32	1.81	3.13	4.89	3.66	3.99	2.95
1367774_at	Gsta3	Glutathione S-transferase A3	1	0.50	−0.31	1.81	2.58	4.11	2.53	2.49	2.31
1384150_at	MID1	Midline 1 (Opitz/BBB syndrome)	3	0.50	0.33	1.79	2.10	4.37	3.53	2.07	2.57
1368725_at	JAG1	Jagged 1	1	−0.50	0.33	1.78	2.47	4.34	3.52	2.72	2.37
1371691_at	RARRES2	Retinoic acid receptor responder (tazarotene induced) 2	1	0.00	−0.33	1.77	2.22	4.44	2.83	2.37	2.79
1375224_at	PHLDA3	Pleckstrin homology-like domain, family A, member 3	1	0.00	−0.33	1.76	4.61	6.21	5.57	4.22	3.29
1378220_at	Rpl39l	Ribosomal protein L39-like	3	0.50	−0.33	1.76	1.57	4.03	2.51	1.83	2.50
1369313_at	FHL2	Four and a half LIM domains 2	1	0.00	−0.33	1.74	2.41	4.26	3.85	2.43	2.26
1388924_at	ANGPTL4	Angiopoietin-like 4	1	0.00	0.33	1.59	3.07	4.39	3.82	2.69	2.61
1373970_at	IL33	Interleukin 33	1	0.49	0.32	1.53	2.43	5.41	4.44	3.58	2.10

**Table 4:** **List of representative pattern-specific genes showing at least 4-fold decrease from the average of their respective controls 24 h after treatment**.

**Probe ID**	**Symbol**	**Entrez gene name**	**Pattern number**	**Control D5W**	**Control NaCl**	**Topotecan followed by D5W**	**Oxaliplatin followed by NaCl**	**Toptecan followed by oxaliplatin**	**Oxaliplatin followed by topotecan**	**NaCl followed by oxaliplatin**	**D5W followed by topotecan**
1397312_at	XPO7	Exportin 7	17	0.00	−0.33	−4.00	−1.98	−4.48	−4.63	−1.84	−4.07
1377079_a_at	PPOX	Protoporphyrinogen oxidase	16	0.00	−0.33	−4.02	−3.42	−4.03	−3.70	−4.17	−3.69
1376118_at	OTUB2	OTU domain, ubiquitin aldehyde binding 2	16	0.00	−0.33	−4.05	−2.76	−4.04	−3.34	−3.29	−3.24
1392512_at	Hist3h2ba	Histone cluster 3, H2ba	17	0.00	−0.33	−4.05	−3.01	−3.41	−3.37	−3.00	−4.93
1389160_at	AHSP	Alpha hemoglobin stabilizing protein	17	0.50	0.33	−4.05	−2.20	−4.79	−4.41	−2.48	−4.98
1383593_at	TMEM56	Transmembrane protein 56	16	0.00	−0.33	−4.07	−4.83	−6.91	−4.69	−6.22	−4.36
1394361_a_at	WNT2	Wingless-type MMTV integration site family member 2	17	0.48	−0.33	−4.08	−2.46	−3.98	−2.70	−3.82	−2.86
1390465_at	RNF123	Ring finger protein 123	17	0.00	−0.33	−4.10	−2.58	−3.55	−2.49	−2.49	−4.20
1370878_at	UROD	Uroporphyrinogen decarboxylase	16	0.00	0.33	−4.13	−3.37	−4.51	−4.31	−3.69	−4.05
1382963_at	ABCB10	ATP-binding cassette, sub-family B (MDR/TAP), member 10	16	0.00	−0.33	−4.13	−3.47	−5.65	−4.44	−3.78	−4.52
1387970_at	SLC38A5	Solute carrier family 38, member 5	17	0.00	0.33	−4.15	−2.74	−4.75	−2.64	−3.75	−4.00
1372523_at	GCLC	Glutamate-cysteine ligase, catalytic subunit	16	0.00	−0.32	−4.24	−3.72	−5.33	−3.70	−4.59	−3.29
1373458_at	BEX4	Brain expressed, X-linked 4	9	0.00	0.33	−4.24	−26.55	−25.17	−23.87	−26.68	−5.09
1392687_at	PIGQ	Phosphatidylinositol glycan anchor biosynthesis, class Q	16	−0.50	0.33	−4.30	−3.53	−6.43	−4.82	−3.90	−3.85
1372086_at	FHDC1	FH2 domain containing 1	17	−0.50	−0.33	−4.31	−1.95	−3.54	−3.33	−1.94	−4.49
1388059_a_at	SLC11A2	Solute carrier family 11 (proton-coupled divalent metal ion transporters), member 2	16	0.00	−0.33	−4.37	−4.18	−4.55	−4.26	−4.36	−4.04
1369305_at	RAB3IL1	RAB3A interacting protein (rabin3)-like 1	17	0.00	−0.33	−4.40	−2.89	−3.91	−3.55	−2.69	−3.49
1367689_a_at	CD36	CD36 molecule (thrombospondin receptor)	15	0.00	0.33	−4.43	−1.92	−4.20	−3.22	−1.65	−3.95
1389507_at	NEDD4L	Neural precursor cell expressed, developmentally down-regulated 4-like, E3 ubiquitin protein ligase	17	0.00	0.33	−4.49	−2.66	−5.11	−4.27	−2.69	−4.30
1387693_a_at	SLC6A9	Solute carrier family 6 (neurotransmitter transporter, glycine), member 9	17	0.00	0.32	−4.50	−2.88	−4.21	−3.84	−2.98	−4.19
1393142_at	CEP70	Centrosomal protein 70kDa	16	0.00	0.33	−4.59	−3.07	−5.11	−4.14	−3.79	−3.79
1395610_at	TSPAN33	Tetraspanin 33	17	−0.49	−0.33	−4.62	−2.70	−4.79	−3.91	−3.61	−4.41
1375896_at	STRADB	STE20-related kinase adaptor beta	17	0.00	0.33	−4.65	−2.49	−4.43	−4.24	−2.62	−4.00
1392695_a_at	UROS	Uroporphyrinogen III synthase	16	−0.50	0.33	−4.77	−6.07	−6.88	−6.03	−6.40	−5.84
1372374_at	CA1	Carbonic anhydrase I	9	0.00	0.33	−4.78	−46.26	−64.49	−67.60	−43.94	−7.59
1387370_at	TMOD1	Tropomodulin 1	17	0.00	0.33	−6.81	−3.78	−7.02	−5.18	−4.02	−7.72
1382618_at	EPB42	Erythrocyte membrane protein band 4.2	17	−0.50	−0.33	−6.84	−3.25	−7.25	−5.11	−3.34	−8.67
1393376_at	SOX6	SRY (sex determining region Y)-box 6	17	0.01	0.31	−6.96	−4.84	−7.27	−6.53	−5.92	−6.58
1383853_at	DYRK3	Dual-specificity tyrosine-(Y)-phosphorylation regulated kinase 3	17	0.00	0.33	−7.14	−2.62	−6.69	−6.60	−2.61	−8.69
1378196_at	SLC43A1	Solute carrier family 43, member 1	17	0.00	−0.33	−7.70	−4.55	−10.31	−9.27	−5.39	−8.31
1369634_at	SLC4A1	Solute carrier family 4	17	0.50	0.33	−7.89	−2.32	−5.68	−3.85	−2.15	−8.21
1371113_a_at	TFRC	Transferrin receptor (p90, CD71)	15	0.00	−0.33	−8.14	−4.93	−15.11	−12.20	−4.97	−10.76
1389398_at	ANK1	Ankyrin 1, erythrocytic	17	0.00	−0.33	−9.38	−4.35	−12.60	−7.48	−4.21	−12.30
1383290_at	SPINT1	Serine peptidase inhibitor, Kunitz type 1	16	−0.50	−0.33	−9.60	−10.69	−13.97	−12.59	−11.11	−10.53
1388998_at	EPB49	Erythrocyte membrane protein band 4.9 (dematin)	17	−0.50	0.33	−10.59	−5.70	−9.84	−8.05	−5.93	−10.33
1381477_at	ANK1	Ankyrin 1, erythrocytic	17	0.00	0.33	−12.66	−6.64	−17.83	−10.27	−7.94	−14.31
1383286_at	PLEK2	Pleckstrin 2	17	0.00	0.33	−14.00	−6.67	−15.65	−11.86	−9.34	−15.86
1387522_at	RHAG	Rh-associated glycoprotein	17	0.00	0.33	−14.97	−5.69	−13.90	−12.95	−6.28	−13.77
1381489_at			17	0.50	0.33	−17.14	−7.25	−22.67	−17.70	−9.82	−16.57
1387100_at	AQP3	Aquaporin 3 (Gill blood group)	16	0.00	−0.33	−17.21	−8.44	−21.00	−13.40	−11.88	−12.18
1389715_at	YPEL4	Yippee-like 4 (Drosophila)	17	0.00	0.32	−19.98	−6.29	−15.03	−13.24	−7.82	−16.63
1386212_at	SPTA1	Spectrin, alpha, erythrocytic 1 (elliptocytosis 2)	17	0.00	−0.33	−26.51	−4.34	−21.38	−11.00	−6.03	−29.53

### Pathway analysis of genes within patterns extracted by EPIG

**Figure 4:**
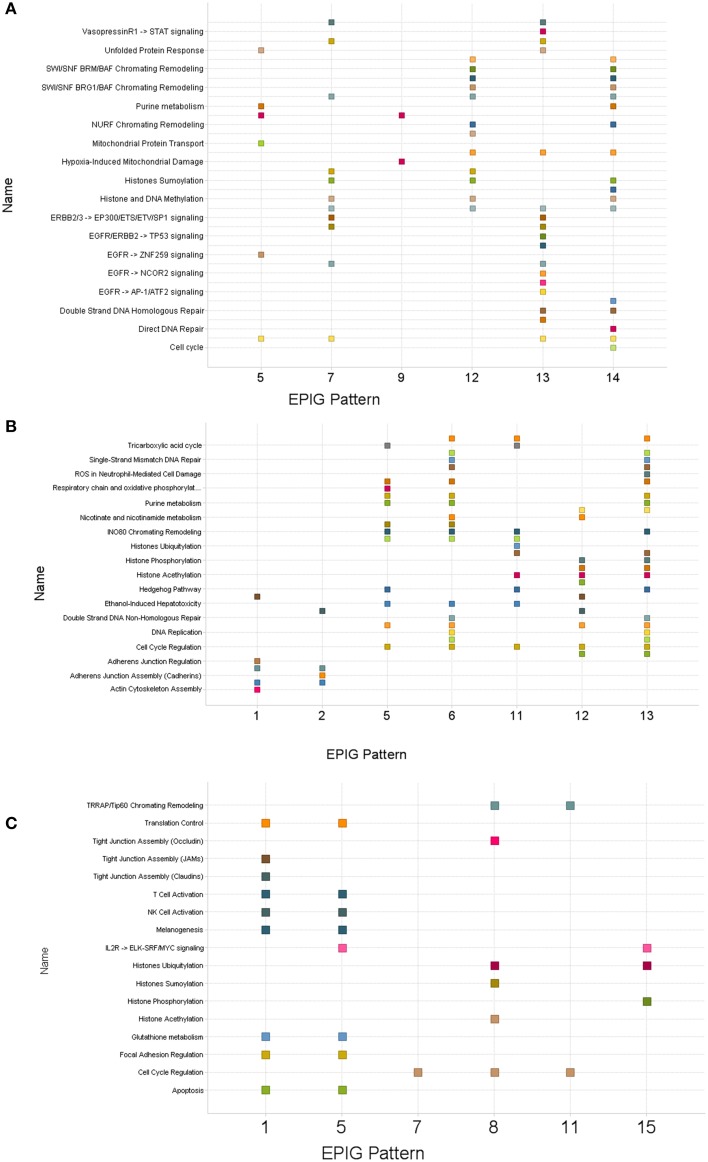
**Scatter plots of pathways enriched in patterns derived from bone marrow collected post-treatment with topotecan, oxaliplatin, or a combination**. Clusters of genes identified by EPIG were then analyzed using Gene Set Enrichment Analysis (GSEA) and Sub-Network Enrichment Analysis (SNEA) algorithms to find enriched pathways. **(A)** One hour post-treatment. **(B)** Six hours post-treatment. **(C)** Twenty-four hours post-treatment. The numbers on the x-axis denote the EPIG pattern which contained the genes that enriched the pathways labeled on the y-axis. A square represents a pathway that was enriched by the genes in the given EPIG pattern.

Gene lists within patterns revealing clear differences between control and treatment groups for each time-point were used to obtain pathway enrichment profiles by gene set enrichment analysis (Subramanian et al., 2005). Supplementary Tables 2–4 are lists (1 h, 6 h and 24 h respectively) of pathways from gene set enrichment analyses carried out against reference pathways, provided by Ariadne, within the designated patterns. Pathways common to multiple patterns were also identified and shown in Figure 4. The pathways commonly enriched in all patterns evaluated at all treatment times were those pathways related to chromatin remodeling and cell cycle regulation. Pathways derived from the lists of genes obtained from bone marrow samples 1 and 6 h after dosing were particularly enriched with pathways related to DNA repair, histone biology, cell cycle regulation, hypoxia, glutathione metabolism, and respiratory and oxidative phosphorylation. These regulatory events provide evidence of target-mediated biology for the drug treatments that can be potentially used as a basis for additional toxicodynamic modeling as early as 1 h after administration of a single dose.

### Differentially expressed genes

Using statistical comparisons of treatments to time-matched controls (absolute fold change >2 and FDR < 0.01), 3304 gene probes in total were detected as differentially expressed (Supplementary Table 5). Principal component (PC) analysis of the gene expression data from the differentially expressed gene probes projected the samples in 3-dimensional space (Supplementary Figure 5) and revealed that PC #1 separates the samples by time and the top 3 PCs grouped the biological replicates by treatment very well.

### Enrichment of biological processes

**Figure 5:**
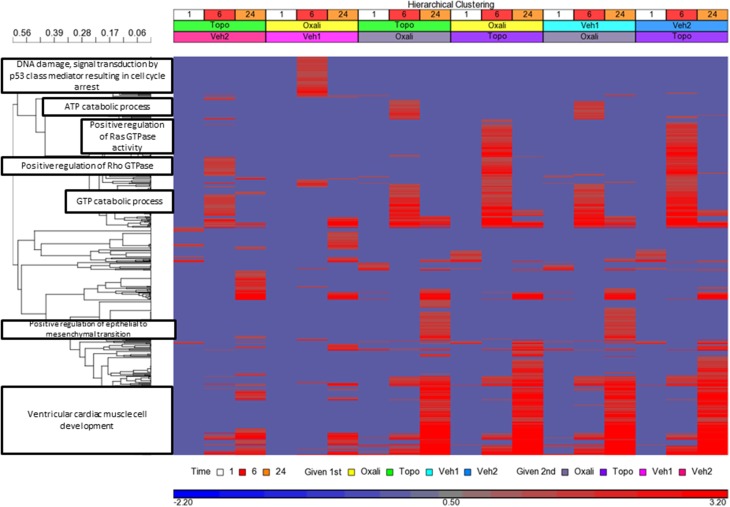
**Clustering of the Enrichment of GO BPs**. Clustering and heatmap based on the −log base 10 (*p*-values) of the 641 Gene Ontology (GO) biological processes (BPs) for each treatment and time point. The GO BPs are on the y-axis, the samples are on the x-axis. The enriched GO BPs were obtained from limma analysis DEGs (absolute fold change >2 and FDR < 0.01). Significance of enrichment was set to *p* < 0.05. Missing values were imputed as 0 (−log base 10(1), i.e., *p* = 1), Clustering of the pathways were performed using Pearson correlation dissimilarity (*r*) and average linkage grouping. Clusters (those with a 1- *r* ≥ 0.9) of GO BP terms (*n* ≥ 30) were labeled according to the node having the maximum number of paths to it within the GO BP subtree directed acyclic graph derived from the terms in the cluster. The color in the legend denotes the significance of enrichment. The more red the heat map color, the more significant the enrichment.

Genes detected as differentially expressed at each time point (Supplementary Table 5) were used to enrich Gene Ontology (GO) biological processes (BPs). There were 641 GO BPs (Supplementary Table 6) significant (*p* < 0.05) from the union of all the lists (Supplementary Tables 7–12) [each one matching the order represented by the treatments in Figure 5]. Figure 5 shows the clustering of the 641 GO BPs terms based on the –log base 10 *p*-values. Clusters (those with a correlation value ≥ 0.9) of GO BP terms (*n* ≥ 30) were labeled according to the node having the maximum number of paths to it within the GO BP subtree directed acyclic graph derived from the terms in the cluster. DNA damage-signal transduction by p53 was highly enriched by the oxaliplatin exposure at the 6 h time point when it was given first but not second. Oxaliplatin given second elicited an ATP catabolic process at 6 h and positive regulation of epithelial to mesenchymal transition at 24 h. Topotecan when given second at 6 h impacted Ras GTPase activity in a positive regulation manner. The GTP catabolic process was enriched at the 6 h time point regardless of the order of administration of topotecan or oxaliplatin. Very few BPs were enriched at the 1 h time points. However, the biological processes highly connected to ventricular cardiac muscle cell development were enriched in a time-dependent manner, maximizing at the 24 h time point.

### Regulation of p53 signaling, apoptosis and cell cycle related genes

**Figure 6:**
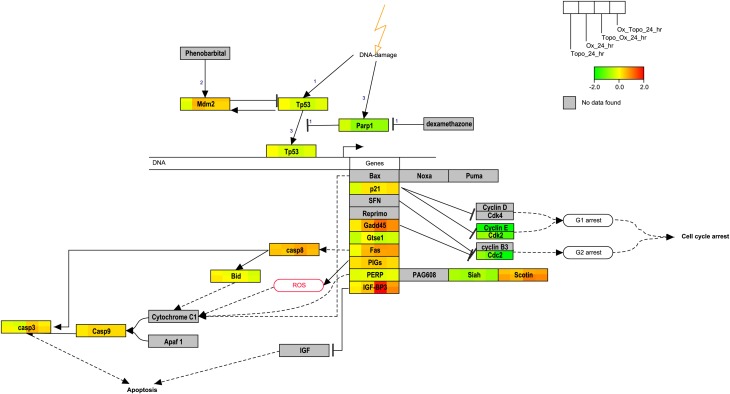
**Overlay of gene expression on the p53 signaling pathway**. The rat p53 signaling pathway is overlaid with the average of the log base 2 ratio values from the replicate samples. Red denotes induction, green repression and yellow no change. Gray indicates that the gene was not mapped. The legend illustrates the segmentation of the genes according to the data from a given treatment.

Pathways related to DNA damage and p53-mediated cell cycle arrest were identified as highly connected and were uniquely enriched in some samples. We then visualized the changes in gene expression within the rat p53 signaling Wikipathway at the 24 h time point in the study (Figure 6). As indicated by the relative expression to time-matched controls, MDM2, GADD45, SCOTIN, CASP8, and IGFB3 were up-regulated whereas the cyclins, p53, SIAH, GTSE1, and PARP1 were down-regulated. Regulation of several genes that also play roles in the apoptosis pathways (Figure 4) and cell cycle regulation (Figure 4) was also observed at the 24 h time point.

### p53 signaling pathway gene regulatory network reconstruction

**Figure 7:**
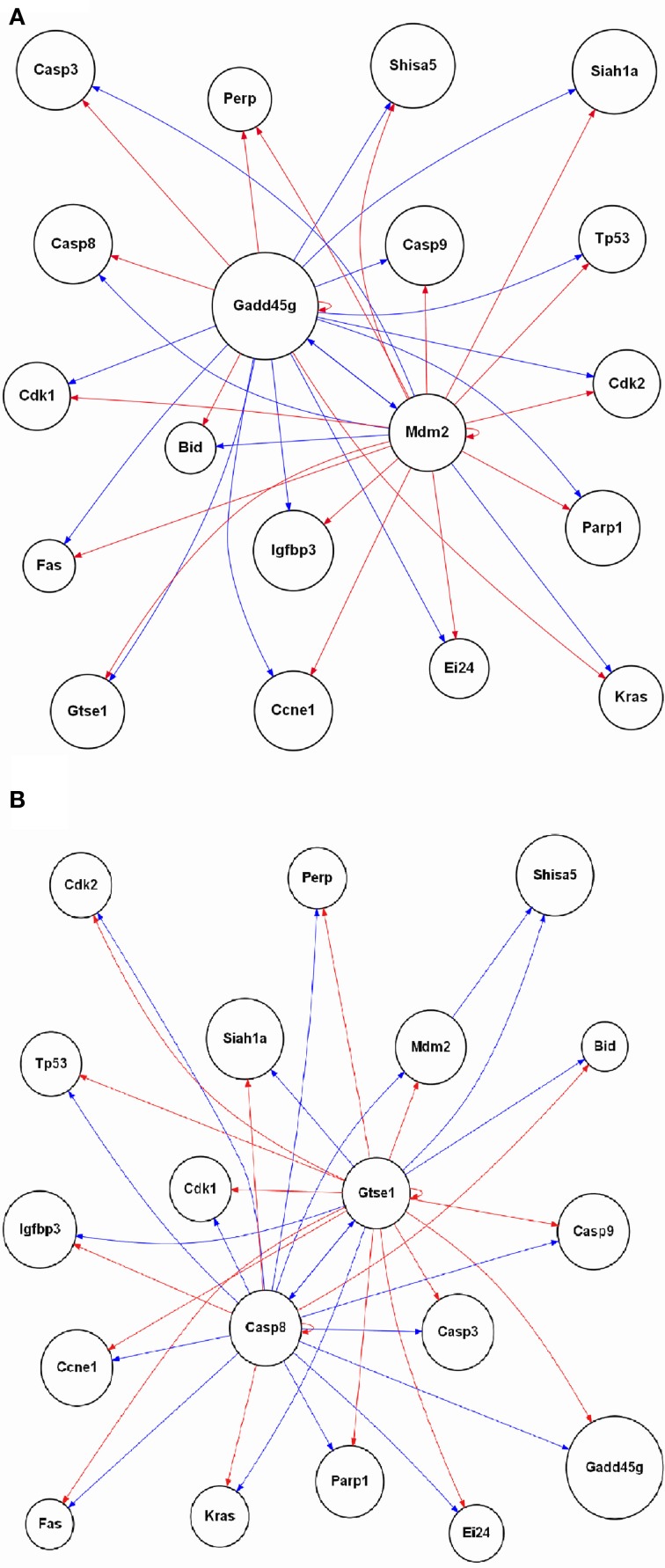
**Reconstruction of the p53 signaling gene regulatory network**. The 19 genes mapped in the rat p53 signaling Wikipathway is used to reconstruct the network based interactions derived from the gene expression data. For **(A)** topotecan followed by oxaliplatin and **(B)** oxaliplatin followed by topotecan, the average of the biological replicates' gene expression data at each time point were used to reconstruct the gene regulatory network depending of the order of administration of the two drugs. A red arrow indicates activation, a blue arrow indicates inactivation. The labeling of the nodes is based on the UniGene symbol.

The enrichment of GO biological processes from the samples where oxaliplatin is given first followed by vehicle for topotecan revealed DNA damage, regulation of p53 signaling transduction (Figure 5). In addition, the overlay of gene expression data on the p53 signaling pathway revealed regulation of key components of the cascade at 24 h (Figure 6). We therefore sort out to reconstruct the gene regulatory network based on the 19 genes mapped to the rat p53 signaling Wikipathway. This would allow us to compare the gene interactions from the time point data in the samples where topotecan is given first followed by oxaliplatin vs. when oxaliplatin is given first followed by topotecan. Using the Gene Regulatory Network Inference (GRNInfer) software with the default setting to control the sparseness and the complexity of the network reconstruction, gene networks based on the average of the four replicate time point studies for each order of administration were revealed (Figure 7). When topotecan is given first followed by oxaliplatin, MDM2 proto-oncogene, E3 ubiquitin protein ligase (MDM2) and GADD45g are central hubs interacting with p53, cyclin-dependent kinases, several cysteine-aspartic acid proteases (CASPs), BID, two CASPs (CASP8 and CASP3) and other components (Figure 7A). On the other hand, when oxaliplatin is given first followed by topotecan, CASP8 and the G-2 and S-phase expressed 1 gene (GTSE1) are the central hubs of the network interacting with p53, the cyclin-dependent kinases, CASP9, Kras, FAS, BID and other components (Figure 7B). Essentially the activation and inactivation shown for components in the networks are caused by different central regulators depending on the order of administration.

## Discussion

This study was conducted to determine whether a single dose rodent study and toxicogenomics profiling informs the potential risk for enhanced bone marrow toxicity for the combined administration of clinically effective chemotherapeutic agents. Genomic profiling is a mature technology that has been strategically and efficiently used in preclinical drug safety assessment to predict safety issues that may be revealed in more lengthy, longer-term studies (Ryan et al., 2008; Huang et al., 2010). In addition, global gene expression data have shown promise in generating hypotheses about early onset “toxicity triggers” (Hamadeh et al., 2010). Topotecan is a particularly difficult drug to use in combination because of the need for dose attenuation when given in combination regimens. Having a method to identify the potential for enhanced bone marrow toxicity when topotecan is given in combination with a second agent would be particularly useful.

During the study, no drug-related adverse clinical signs were observed for any animals treated with topotecan or oxaliplatin alone or in combination. Histopathological evaluation of bone marrow established the presence of drug-related lesions in bone marrow (hypocellularity) in animals administered topotecan or oxaliplatin. Although our preclinical results highlight the need to consider the sequence of administration in a clinical protocol, our histopathological findings were not consistent with the sequence dependency of the severity of bone marrow toxicity that was reported in clinical studies of cisplatin and topotecan (De Jonge et al., 2000). In clinical trials, both neutropenia and thrombocytopenia were more severe when cisplatin was administered prior to topotecan and there were significantly lower absolute neutrophil count nadirs and percentage decrements in neutrophil and platelet counts with this sequence. Our results are consistent with the observation that oxaliplatin exhibits a favorable toxicity profile with a substantially lower incidence and severity of nephrotoxicity, ototoxicity, and myelosuppression.

Topotecan and other anticancer agents may cause DNA damage via mechanisms other than direct binding to DNA replication machinery. For example, camptothecin and analogs have been reported to induce apoptosis through a mechanism involving reactive oxygen species and oxidative stress pathways (Li et al., 2009). Similarly, pretreatment of mice with the anti-oxidant quercetin was reported to reduce topotecan-induced genotoxicity and cytotoxicity (Bakheet, 2011). Quercetin also reduced oxidative stress markers in topotecan treated bone marrow cells (Bakheet, 2011). Thus, it is feasible that the early effects of topotecan we observed in bone marrow are related to oxidative stress. Although it is clear that most of the *in vivo* biology for topotecan would be expected to be mediated by TOP1, the observed enrichment of genes related to ROS neutrophil-mediated cell damage and hypoxia-induced mitochondrial damage pathways, support additional mechanistic possibilities.

Traditional gene expression analysis has used two samples statistical tests for each comparison of interest or analysis of variance (ANOVA) model when the data are from studies with a factorial design. Upon selecting differentially expressed genes (DEGs), the next step is usually pooling the lists of DEGs to cluster analyze the data for visualization of patterns of gene expression across the samples. A limitation to this strategy is that the process of selecting the genes before detecting the patterns can omit genes that have salient expression profiles correlated across the samples. This caveat is of major concern when analyzing expression data comprised of genes whose expression are altered at low drug exposures or perturbed only for a short duration. The EPIG approach (Chou et al., 2007) was developed specifically for toxicogenomics and other series type studies where the algorithm uses an ANOVA-like statistical evaluation of the patterns of gene expression but also takes into account correlation of genes within an expression pattern. In the EPIG two-step approach, all significant patterns in the data are extracted first, followed by categorization of gene expression profiles.

Application of the EPIG method to our microarray data and subsequent pathway and gene regulatory network analyses allowed identification of genes, pathways and regulatory interactions that appear to represent promising biomarker signatures for bonemarrow toxicity. Pathways related to p53 signaling, DNA repair, histone biology, cell cycle regulation, hypoxia, glutathione metabolism, and respiratory and oxidative phosphorylation reflect the biological effects of either treatment on the bone marrow. The considerable enrichment of pathways involved with DNA repair and chromatin remodeling is remarkably well-aligned with the biological mechanisms and downstream effects of topotecan. The interplay between chromatin remodeling and DNA repair factors is infrequently discussed in relation to DNA damage response mechanisms of the bone marrow. Our analysis highlights a meaningful relationship between chromatin remodeling complexes and mechanisms of bone marrow toxicity and repair that warrants further investigation.

An interesting biological response pathway of genes related to tissue injury was derived from our analysis of co-expressed genes (Supplementary Table 1). Regulation of chondrogenesis, bone repair, and differentiation was identified for some of the genes that were profoundly increased in response to combination treatment when compared to individual treatments. These genes were matrix metallopeptidase 12 (MMP12); transgelin (TAGLN, SM22α); cyclin D1 (CCND1); serpin peptidase inhibitor, clade H (heat shock protein 47), member 1 (collagen binding protein1) (SERPINH1); tenascin C (TNC); ADAM metallopeptidase with thrombospondin type 1 motif, 9 (ADAMTS9); and bone morphogenetic protein 2 (BMP2). TNC is an extracellular matrix glycoprotein that is specifically and transiently expressed upon tissue injury. SERPINH1 functions as a molecular chaperone during collagen synthesis and maturation (Nagata, 1998; Lamande and Bateman, 1999; Razzaque and Taguchi, 1999; Hendershot and Bulleid, 2000). Upon tissue damage, TNC regulates a wide variety of pathways that mediate both inflammatory and fibrotic processes, enabling effective tissue repair (Truong et al., 1996; Chiquet-Ehrismann and Chiquet, 2003; Midwood and Orend, 2009). TAGLN is a shape change sensitive 22 kDa actin-binding protein of the calponin family that may regulate conversion of adult bone marrow-derived mesenchymal stem cells into smooth muscle cells (SMCs) and is an early marker of smooth muscle differentiation (Lawson et al., 1997). Expression of ADAMTS9 was shown to be up-regulated during chondrogenic differentiation of human mesenchymal stem cells (Boeuf et al., 2012).

Gene profiling of bone marrow environment cells revealed distinct expression profiles for genes encoding for ADAMs and their inhibitors (Bret et al., 2011). In the current study, all cells expressed ADAMTSs genes at a low level, with the exception of bone marrow stromal cells. BMP2 plays an essential role in chondrocyte proliferation and maturation during endochondral bone development (Shu et al., 2011). Similarly, MMPs are required for both endochondral and intramembranous ossification during bone repair and it is likely that this gene response is a sensitive indicator of initial degradation of extracellular matrix. These gene responses are similar to microarray studies that identified gene responses during stages of bone marrow-ablation-induced bone regeneration (Wise et al., 2010). Taken together, the observed gene responses may represent a unique biomarker panel for bone marrow that will flag early tissue damage onset followed by chondrogenesis and intramembranous regeneration processes. The 11- and 7-fold increases in MMP12 and TAGLN observed 24 h post-treatment with topotecan compared with a 54- and 17-fold increase when topotecan treatment is given with oxaliplatin might represent an additive response for the combination. The effect of treatment combinations on these responses likely reflects a change in the extent of damage and response of the bone marrow to injury.

Among the genes showing the most profound decreases in response to all treatments are several genes for proteins regulating shape and hemolysis of erythrocytes. We noted a 26-fold decrease in spectrin, alpha, erythrocytic 1 (elliptocytosis 2) (SPTA1). Mutations in spectrin genes that render red cells deficient in spectrin are associated with abnormal cytoskeletal architecture making erythrocytes susceptible to hemolysis (Kakhniashvili, 2001; Broderick and Winder, 2005). A similar contribution to actin dynamics in platelets has been elucidated for pleckstrin-2 (PLEK2) (Lian et al., 2009), and we report a 14-fold decrease in PLEK2 mRNA in response to topotecan alone. In addition, a 9-fold decrease was noted in Ankyrin 1 (ANK 1), an erythrocyte membrane protein that is defective in many patients with hereditary spherocytosis, a common hemolytic anemia. Taken together our results demonstrate a clear connectivity between the most profoundly affected genes and the clinical adverse effect profile for topotecan. For example, a recent clinical study designed to determine the dose of weekly oral topotecan allowing safe administration in patients with recurrent gynecologic malignancies, 13 (11.1%) doses of drug were held because of anemia in 8 patients, neutropenia in 7, or thrombocytopenia in 2 (Von Gruenigen et al., 2012).

This study reports gene response data in bone marrow post-treatment with topotecan or oxaliplatin alone and in combination following a single administration. There are no comparable gene expression studies on the effect of the combination of the two agents used here, but one study, based on 8 mg/kg oxaliplatin in rat bone marrow at 24 h, was found in a public data source. That public (yet unpublished) study is deposited in the Drug Matrix 8.0 database (Ganter et al., 2005) hosted by the NIEHS/NTP at https://ntp.niehs.nih.gov/drugmatrix/index.html. One published study compares the effect of oxaliplatin in ovarian cancer spheroids (L'Esperance et al., 2008). This underscores the uniqueness and novel aspect of our combination study. Supplementary Figures 6, 7 show the paucity of overlap of the oxaliplatin DEGs from our current study and those from the aforementioned public studies (Drug Matrix bone marrow and ovarian cancer spheroids respectively). Caution is needed in interpreting these comparisons due to the differences in the doses administered, the target cell type/tissue, the array platforms, significance test and the annotations based on gene symbol to compare results. We provide a comprehensive list of genes from our microarray analysis of bone marrow taken at various time points after treatment of rats to enable additional analysis and hypothesis generation (http://tools.niehs.nih.gov/cebs3/ui/). Although we will continue to analyze these data for biological response pathways and mechanistic pathways for toxicity, we publish these data to encourage other investigators to employ alternative analysis methods and propose additional relationships relevant to the observed bone marrow responses and treatments.

The use of a single dose study using genomic endpoints as a measure of enhanced bone marrow toxicity is exciting for several reasons. This approach could be incorporated into a mechanism-based risk evaluation and a single dose administration can be readily incorporated as an *in vivo* or *in vitro* screening paradigm. For example, attempts to validate *in vitro* bone marrow systems could use these data to explore and identify molecular anchors that translate from *in vitro* to *in vivo*. Second, this proof of concept revealed that the molecular responses in bone marrow toxicity is much earlier than previously documented preclinical histopathological observations of topotecan toxicity. Examining the acute response of a target organ to chemotherapy and obtaining a molecular footprint associated with this response in combination with a targeted agent can guide us along new avenues of investigation about mechanisms of target-mediated toxicity. At this point, these data represent qualitative information that is relevant to understanding mechanisms of toxicity. The initial phenotypic anchoring of these data with additional endpoints may ultimately provide a more comprehensive understanding of pathways underlying bone marrow toxicities. Indeed, other reports have highlighted the potential use of toxicogenomics data to enable integrative risk assessment and biomarker identification (Ellinger-Ziegelbauer et al., 2011; Matheis et al., 2011). We plan to apply this approach to other target organs and additional anticancer drug combinations to examine the broader implications of this strategy.

### Conflict of interest statement

The authors declare that the research was conducted in the absence of any commercial or financial relationships that could be construed as a potential conflict of interest.
