# Supplementary figures and images for: Toxicogenomics profiling of bone marrow from rats treated with topotecan in combination with oxaliplatin: a mechanistic strategy to inform combination toxicity
Source: Front Genet. 2015 Feb 12;6:14. doi: 10.3389/fgene.2015.00014 (PMC4325931; doi:10.3389/fgene.2015.00014)

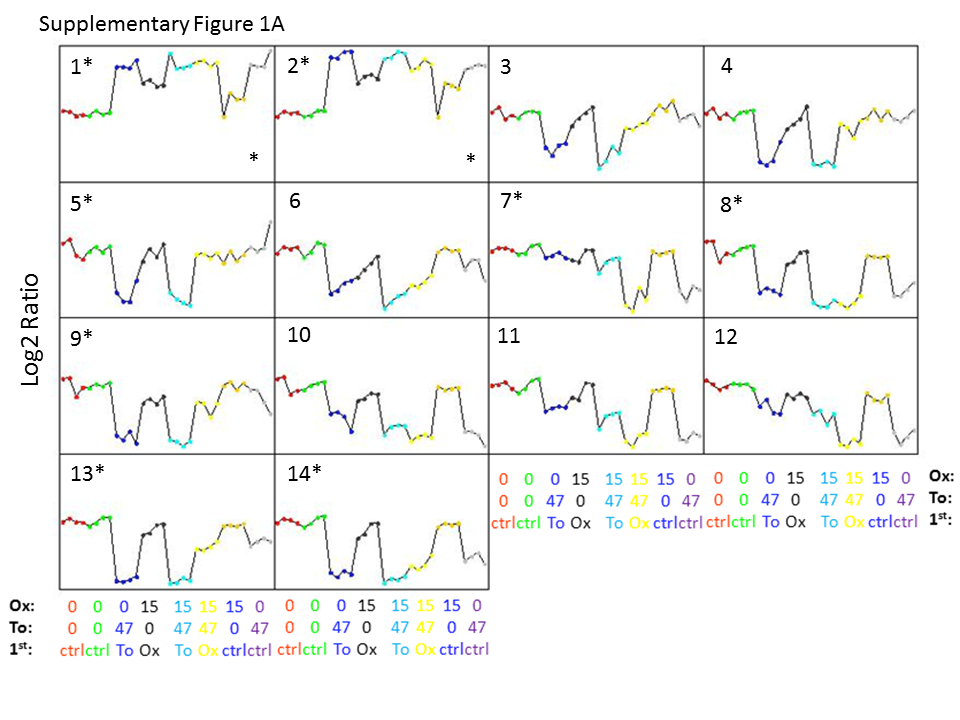

Supplement: Supplementary file 1 [file Presentation1.ZIP › Supplementary Material/Supplementary_Image_1_A.TIF]

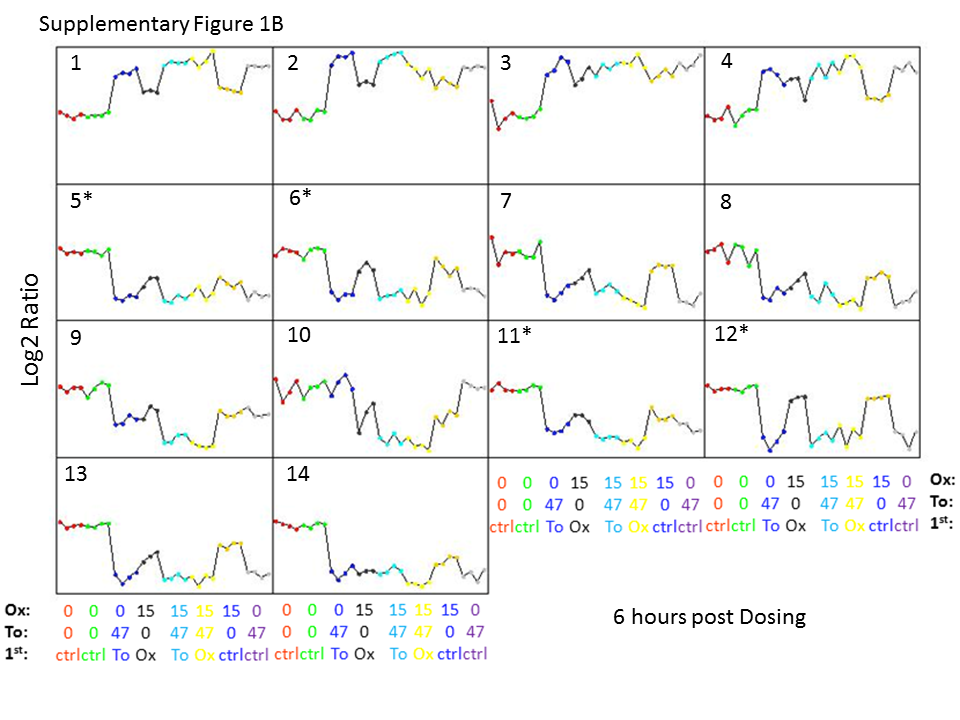

Supplement: Supplementary file 1 [file Presentation1.ZIP › Supplementary Material/Supplementary_Image_1_B.TIF]

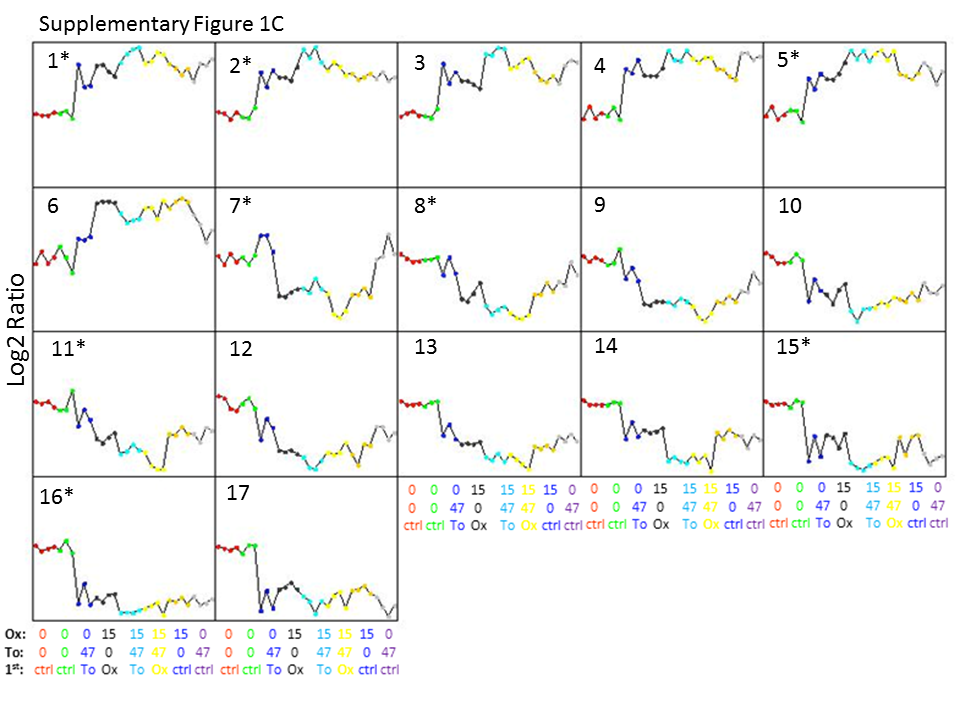

Supplement: Supplementary file 1 [file Presentation1.ZIP › Supplementary Material/Supplementary_Image_1_C.TIF]

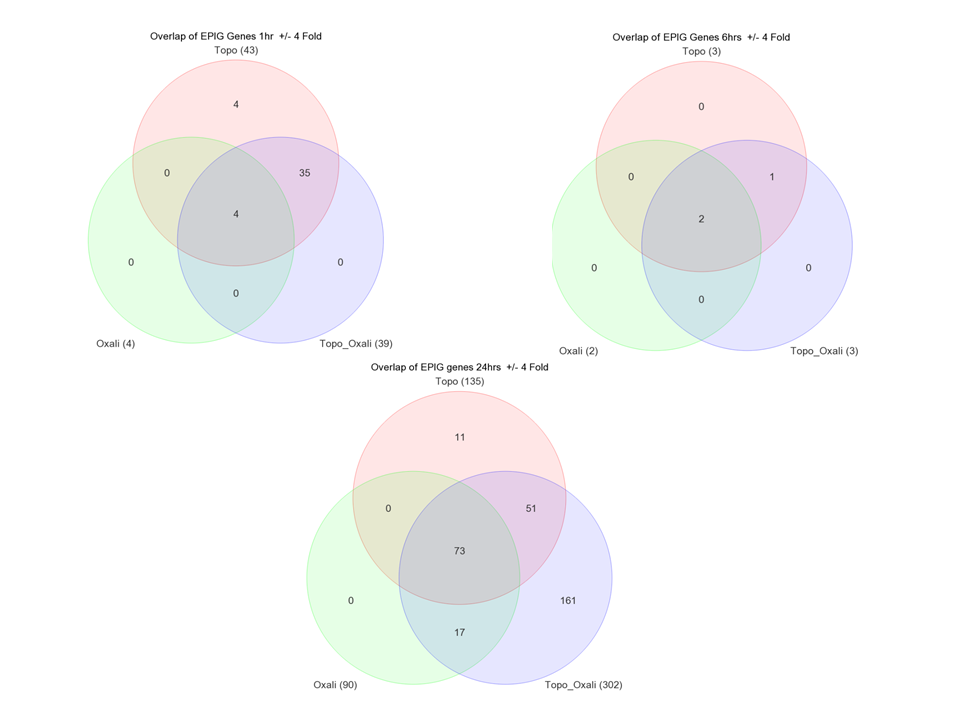

Supplement: Supplementary file 1 [file Presentation1.ZIP › Supplementary Material/Supplementary_Image_2.TIF]

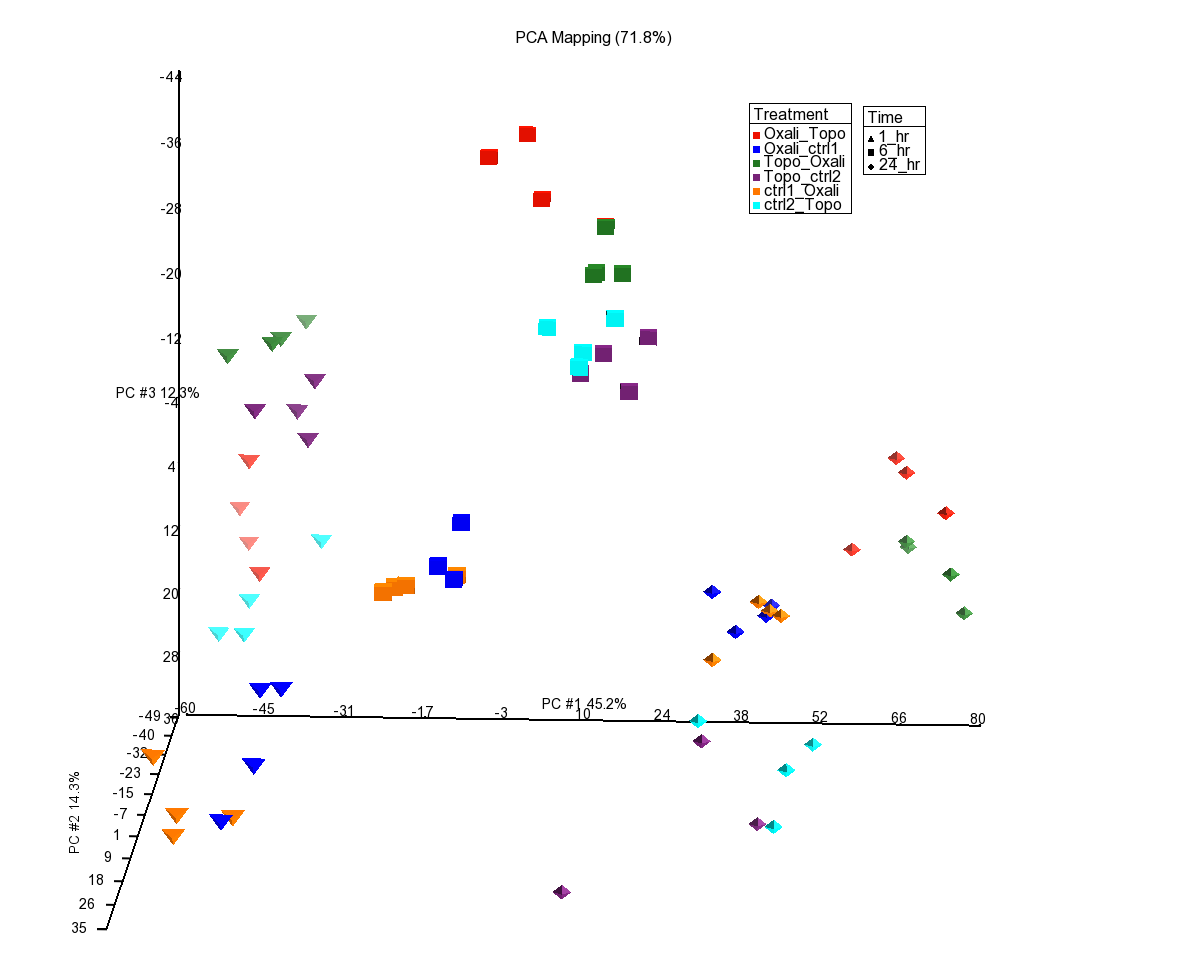

Supplement: Supplementary file 1 [file Presentation1.ZIP › Supplementary Material/Supplementary_Image_3.TIFF]

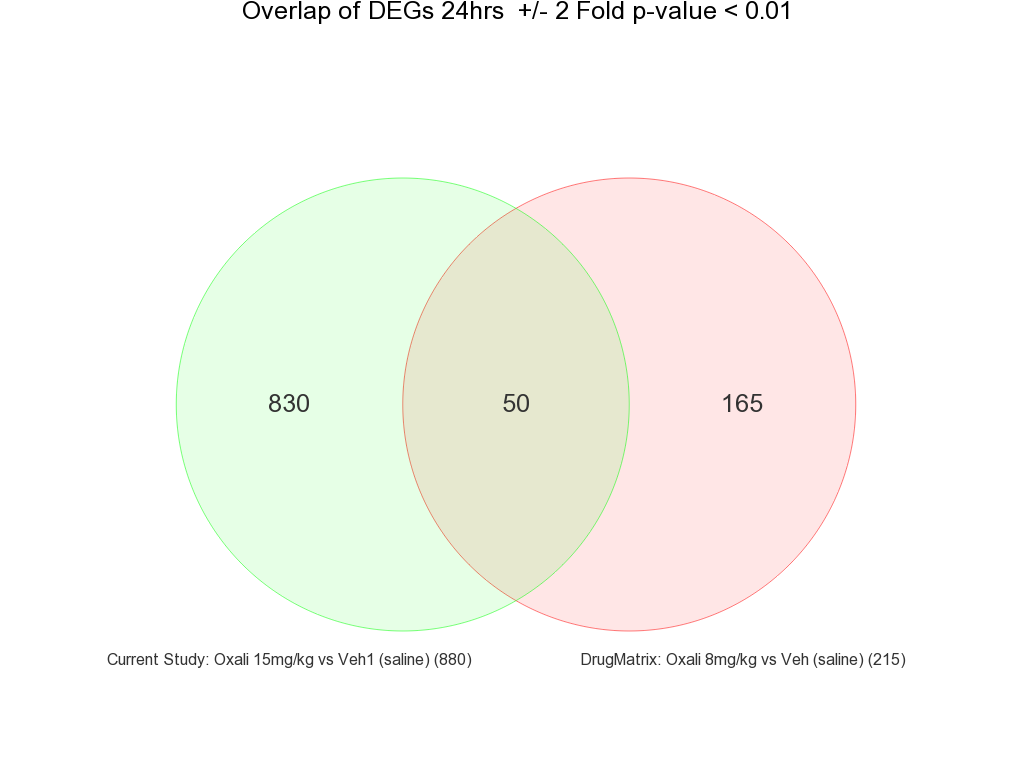

Supplement: Supplementary file 1 [file Presentation1.ZIP › Supplementary Material/Supplementary_Image_4.TIFF]

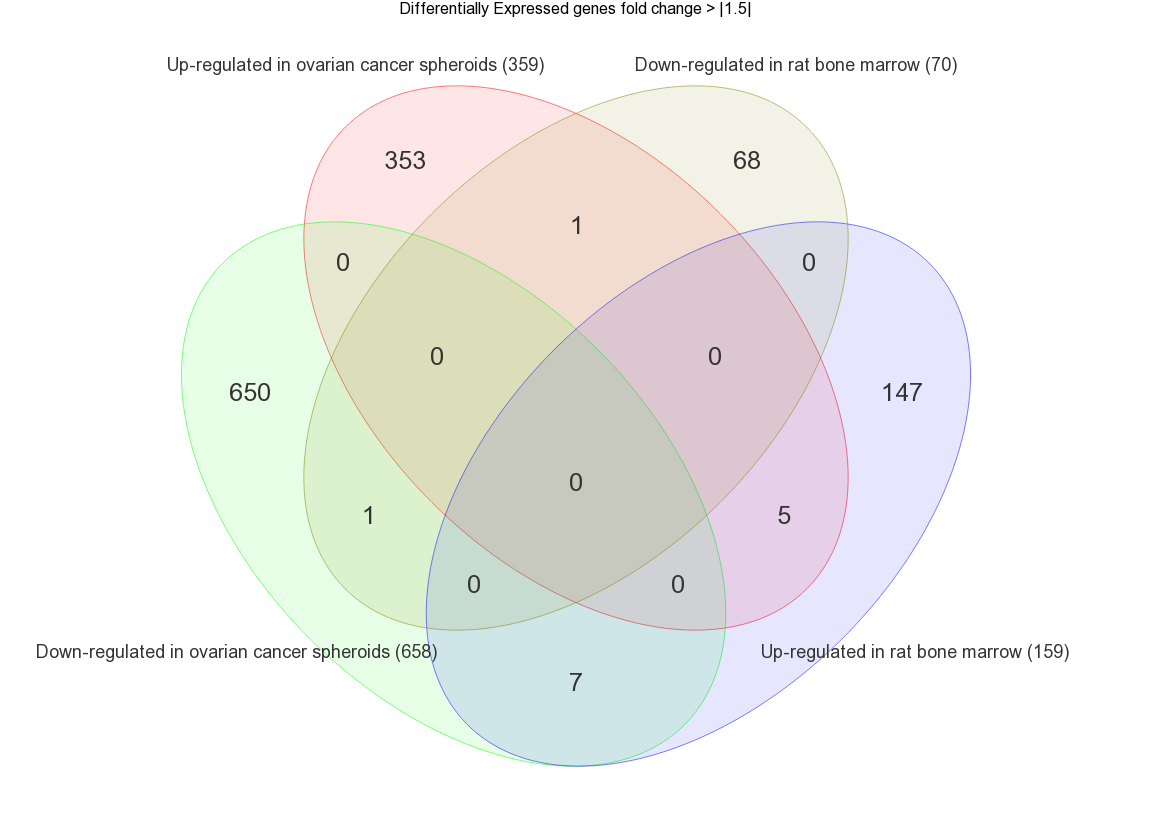

Supplement: Supplementary file 1 [file Presentation1.ZIP › Supplementary Material/Supplementary_Image_5.TIFF]
